# *Pitx3* and *En1* determine the size and molecular programming of the dopaminergic neuronal pool

**DOI:** 10.1371/journal.pone.0182421

**Published:** 2017-08-11

**Authors:** Willemieke M. Kouwenhoven, Lars von Oerthel, Marten P. Smidt

**Affiliations:** Swammerdam Institute for Life Sciences, University of Amsterdam, Amsterdam, the Netherlands; Rutgers University, UNITED STATES

## Abstract

Mesodiencephalic dopaminergic (mdDA) neurons are located in the ventral midbrain. These neurons form the substantia nigra (SNc) and the ventral tegmental area (VTA). Two transcription factors that play important roles in the process of terminal differentiation and subset-specification of mdDA neurons, are paired-like homeodomain transcription factor 3 (*Pitx3*), and homeobox transcription factor Engrailed 1 (*En1*). We previously investigated the single Pitx3KO and En1KO and observed important changes in the survival of mdDA neurons of the SNc and VTA as well as altered expression of pivotal rostral- and caudal-markers, *Ahd2* and *Cck*, respectively. To refine our understanding of the regional-specific relationships between *En1* and *Pitx3* and their (combined) role in the programming mdDA neurons on the rostral-to-caudal axis, we created double *En1*^*tm1Alj/tm1Alj*^*;Pitx3*^*gfp/gfp*^ (En1KO;Pitx3GFP/GFP) animals. Here we report, that in absence of *En1* and *Pitx3*, only a limited number of mdDA neurons are present at E14.5. These mdDA neurons have a rudimentary dopaminergic cell fate, as they express *Nurr1*, *Pbx3* and *Otx2* but have lost their rostral or caudal subset identity. Furthermore, we report that the expression of *Cck* depends on *En1* expression, while (in contrast) both *Pitx3* and *En1* are involved in the initiation of *Ahd2* expression. Thus we reveal in this manuscript that regulated levels of *Pitx3* and *En1* control the size and rostral/caudal-identity of the mdDA neuronal population.

## Introduction

The neurons of the substantia nigra (SNc) and the ventral tegmental area (VTA) originate in from the di- and mesencephalon, and as such are called mesodiencephalic dopaminergic (mdDA) neurons. These neurons are important enforcers of movement and motivation, and are targeted in neurodegenerative pathologies, such as Parkinson's Disease (PD). Interestingly, post-mortem tissue of PD patients revealed that mdDA neurons of the SNc are more vulnerable to cell loss (~80% of the SNc neurons are lost), while the mdDA neurons of the VTA are spared more (~50% loss) [1,2]. In order to enhance the understanding of subset specific vulnerability of mdDA neurons, many efforts have been made to understand the molecular similarities and differences between SNc and VTA neurons. Large rodent-based micro-array studies determined that the difference in molecular profile between the SNc and VTA is smaller than 3% [3–5]; reviewed in [6]. Several studies from our group contributed to the quest to define molecular profiles for different mdDA subsets already during embryonic development [7–10]. Recently, elegant transcriptomic studies identified these molecular profiles in single-cellular resolution in early post-natal tissue [11], as well as during embryonic development of both murine and human tissue [12].

Embryonic mdDA neurons that mature into the SNc can be distinguished from mdDA neurons that develop into the VTA, based on their anatomical position and molecular profile [10]. An important marker of the rostrolateral mdDA neurons is aldehyde dehydrogenase family 1 (*Ahd2/Aldh1a1*) [11,13] which is involved in the metabolism of retinoic acid (RA) out of retinol, and as such has a role during neuronal development in processes of differentiation, and survival [14,15]. Moreover, aldehyde dehydrogenases are required for the metabolism of 3,4-dihydroxyphenylacetaldehyde (DOPAL), which is a toxic metabolyte of dopamine [16]. The relevance of *Ahd2* as a marker for an mdDA neuronal subset was strengthened by the report that the ventral tier of the human SNc, which consists of *Ahd2*-positive mdDA neurons, is the most sensitive to premature cell death in PD [1,17]. Remarkably, a recent study using a mouse model for over-expressing α-synuclein showed that *Ahd2*-negative neurons of the mdDA neurons were more vulnerable for neurodegeneration, suggesting a protective role for *Ahd2* [17]. In contrast, mdDA neurons that will develop into the VTA are characterized by the expression of Cholecystokinin (*Cck*) and are located caudomedially at embryonic day (E)14.5 [13]. *Cck* is a neuropeptide, that has been linked to dopaminergic-mediated pathologies such as schizophrenia and addiction, and its receptors are expressed in the nucleus accumbens and the VTA [18]. Furthermore, *Cck* exerts a neuroprotective role on cholinergic neurons after a basal-forebrain lesion was introduced in rats [19]. Recently, such a neuroprotective role for *Cck* was also described in rat hippocampal cultured neurons. A 14-day supplementation of Cck-8S to adult rats significantly reduced TUNEL-activity and increased the number of KI67-positive neurons in the granular layer of the hippocampus [20]. Whether *Cck* also fulfills a neuroprotective role in mdDA neurons is yet unclear.

Three transcription factors that play important roles in the process of terminal differentiation and subset-specification of mdDA neurons, are the orphan nuclear hormone receptor *Nurr1* (*Nr4a2*), paired-like homeodomain transcription factor 3 (*Pitx3*), and homeobox transcription factor *Engrailed 1* (*En1*). *Nurr1* expression starts in the midbrain at E10.5 and continues to be expressed in mdDA neurons into adulthood [21]. Loss of *Nurr1* results in the ablation of *Th*, *Vmat2*, *Dat*, but *Pitx3* and *En1* expression remain unaffected [21–24]. *Nurr1* initiates the expression of most of the DA genes in cooperation with *Pitx3*, as both transcription factors interact with similar dopaminergic gene regulatory transcription complexes [22]. *Pitx3* is selectively expressed in all mdDA neurons from E11.5 onward [25], but in *Pitx3*-ablated animals mainly the mdDA neurons of the SNc are affected. Furthermore, *Pitx3* is pivotal for the expression of *Ahd2* (through binding to a promoter region of *Ahd2*), as its expression is lost in *Pitx3*-deficient animals [26]. Importantly, in absence of *Pitx3*, *Nurr1* expression remains unchanged, while *En1* is upregulated [22,27]. The homeobox transcription factor *En1* is expressed in mid- and hindbrain from E8 onwards [28], and *En1*-null mice are characterized by cell loss of the entire SNc, as well as the majority of the VTA [13]. *Nurr1* expression is unaltered in *En1*-ablated animals, but the expression of *Pitx3* is diminished, suggesting that *Pitx3* and *En1* modulate each others expression levels. Interestingly, the regulation of *En1* and *Pitx3* upon each other is markedly different between the rostral and the caudal subsets, and the absence of either *Pitx3* or *En1* affects each subset differently [13].

To refine our understanding of the regional-specific relationships between *En1* and *Pitx3* and their (combined) role in mdDA neuronal programming, we created the double *En1*^*tm1Alj/tm1Alj*^*;Pitx3*^*gfp/gfp*^ (En1KO;Pitx3GFP/GFP) animal. Here we report that in absence of *En1* and *Pitx3*, only a limited number of mdDA neurons are present at E14.5, underlining the necessity of *Pitx3* and *En1* in the generation and/or survival of embryonic mdDA neurons. The remaining mdDA neurons still express *Nurr1*, *Pbx3*, and *Otx2* but have lost their rostral (*Ahd2*+) or caudal (*Cck*+) subset identity. Additionally, the diminished expression of *Cck* illustrates its dependence on *En1* activation, while (in contrast) both *Pitx3* and *En1* are involved in the initiation of *Ahd2* expression. Thus, these data further substantiate the notion that *En1* and *Pitx3* determine the size and subset-specificity of the mdDA neuronal population.

## Methods

### Animals

All animals experimentation was supported and granted by the Animals experimentation committee of the University of Amsterdam according national and international legislation. Embryos were isolated at embryonic day (E)14.5, considering the morning of detection of the vaginal plug as E0.5. Tissue was isolated at post-natal day (P)0 (day of birth), immediately after birth, before lethality set in.

*Pitx3*^*gfp/gfp*^ animals, in which the *Pitx3* gene is substituted by a GFP allele [29], were inter-crossed with *En1*^*tm1Alj/+*^ animals to breed the 'intermediate genotype' *En1*^*tm1Alj/+*^;Pitx3^*gfp/gfp*^. These animals were bred to generate litters that included *En1*^*+/+*^;*Pitx3*^*gfp/gfp*^, *En1*^*tm1Alj/+*^;*Pitx3*^*gfp/gfp*^ and *En1*^*tm1Alj/tm1Alj*^;*Pitx3*^*gfp/gfp*^. Genotyping on the *Pitx3*^*gfp*^ allele and the *En1*-mutant allele were performed as described previously [30]. All mice are readily present in our lab.

Male and female mice a separately housed in IVC units (“Tecniplast Smartflow”) in type 2 cages. Breeding was performed in regular type 2 cages. The mice are housed with “Lignocel” bedding with soap-less tissues as cage-enrichment (“Kleenex”) with ad-lib access to “SDS-special diet” for food and ad-lib water. Animals are cared for on a daily basis according to rules and regulation of dutch and EU law. Animals were sacrificed by exposure to CO2/O2: 70%/30% for 3 minutes and then by cervical dislocation or decapitation according rules and regulation of Dutch and EU law.

### Fluorescence-activated cell sorting (FACS) and dissection

Midbrains and rostral hindbrains were dissection in L15-5% Fetal Calf Serum (Gypko). Dissociation and sorting of mid-hindbrains were performed as described previously [13,27]. In short, freshly isolated tissue were dissociated using a Papain dissociation system (Worthington). Cells were sorted on a BD FACS Aria III using previously described settings [27] and collected in Trizol-LS (Invitrogen).

### Quantitative PCR (qPCR)

Relative expression levels were determined by qPCR real-time PCR (Lightcycler) using the QuantiTect SYBR Green PCR LightCycler Kit (QIAGEN) according to the manufacturer’s instructions. For each reaction 0.1 ng (FAC-sorted neurons) total RNA was used as input. Primer pairs were previously published [27].

### Fluorescent immunohistochemistry

Embryos were fixed in 4% paraformaldehyde in PBS, cryoprotected in 30% sucrose in PBS and subsequently stored at -80°C. Sagittal sections (16 μm) were cut on a cryostat, after which they were washed with PBS and blocked in 4% Fetal Calf Serum (FCS) in THZT (50 mM Tris-HCl pH 7.6, 0.5 M NaCl, 0.5% Triton) or PBS-T (0.5% Triton). After another wash treatment with TBS, sections were incubated overnight at 4°C with primary antibody in THZT. Sections were washed three times (PBS) the following morning and incubated for minimally 2 hours at room temperature with secondary antibody in PBS, followed by wash treatment with PBS. DAPI staining was performed (1mg/ml 1:5000) for 5 min, after which section were washed with PBS. Finally, sections were embedded with Fluorsave.

Primary antibodies that were used: Rabbit-α-Th (Pelfreeze, 1:1000), Chicken-α-GFP (Abcam, 1:1000). Secondary antibodies that were used: Goat-α-Rabbit Alexa 555 (1:1000), Goat-α-Chicken Alexa 488 (1:1000), all Invitrogen.

### *In situ* hybridization

*In situ* hybridization was performed as described previously [24]. The Digoxigenin-labeled probe for *Nurr1* was used as described [22]

### Statistical analysis

Values are expressed as means ± standard error of the mean. Comparisons were made using, two-tailed Student's t-test. P<0.05 was considered significant, and indicated using an *, additionally ** = P<0.01; *** = P<0.001.

## Results

### Generation of double En1KO;Pitx3GFP/GFP animals

**Fig 1:**
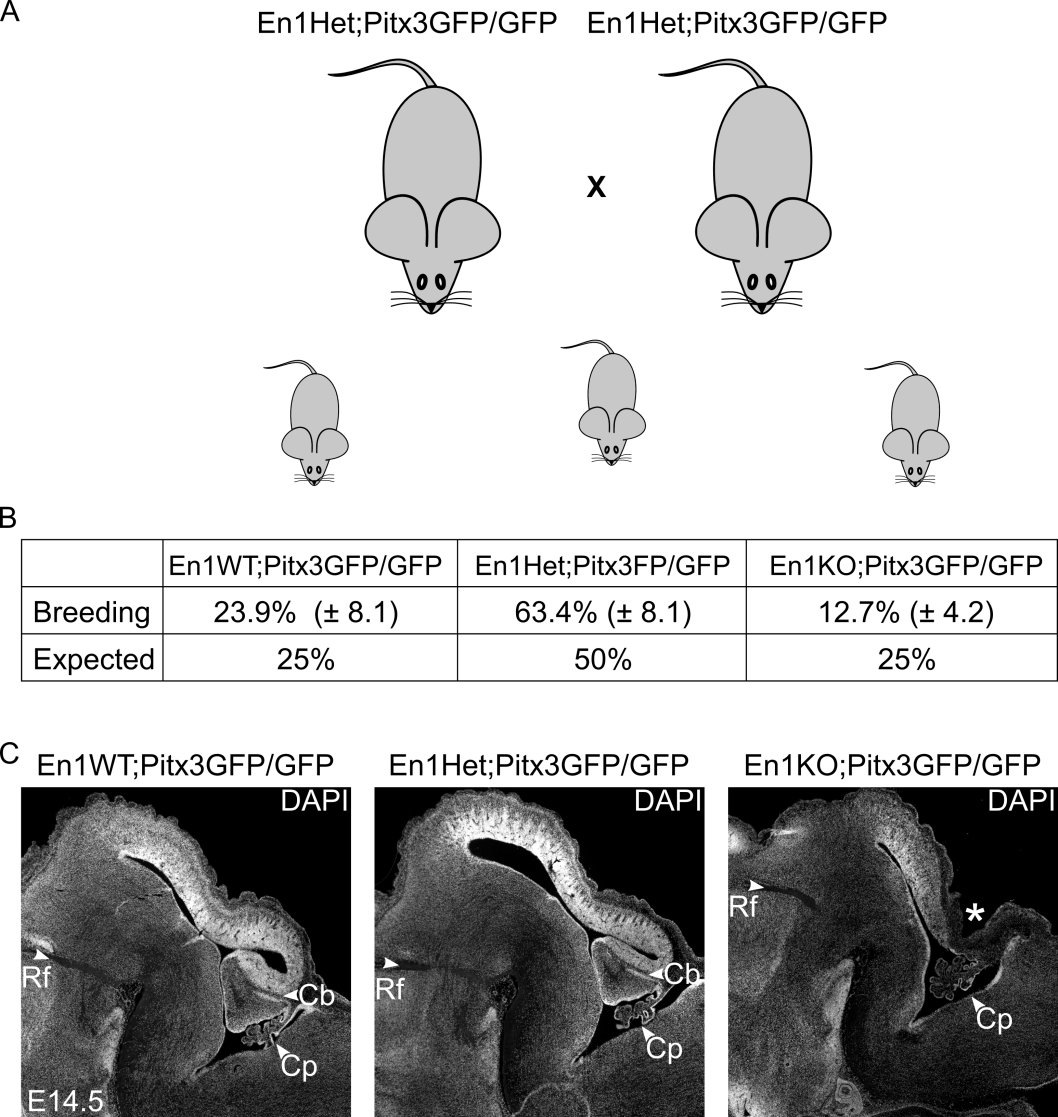
Breeding schedule and outcome of the generation of En1KO;Pitx3GFP/GFP animals. (A) En1Het;Pitx3GFP/GFP animals were inter-crossed to generate a litter that includes three genotypes: En1WT;Pitx3GFP/GFP, En1Het;Pitx3GFP/GFP, and En1KO;Pitx3GFP/GFP. (B) The presence of double En1KO;Pitx3GFP/GFP animals occurred below (Mendelian) predicted chance level (12.7% versus 25%). (C) DAPI staining at E14.5 reveals that in En1WT;Pitx3GFP/GFP and En1Het;Pitx3GFP/GFP animals the cerebellar *anlage* is correctly developed (Cb), whilst it is absent in the double En1KO;Pitx3GFP/GFP (*). (Rf: retroflexus, Cb: cerebellum, Cp: choroid plexus).

Our research group previously investigated single Pitx3KO and En1KO animals to better understand the role of *Pitx3* and *En1* during the development of mdDA neurons. We showed that in absence of *Pitx3* the SNc is absent [31] and in absence of *En1* we observed a dramatic loss of *Th*-expressing cells, that included mdDA neurons from both the SNc and VTA [13]. Moreover, we reported that *Pitx3* and *En1* heavily modulate each other’s expression levels (through transcriptional control, transcriptional complex composition and/or protein-protein interactions) [13]. To further improve our understanding of the regulatory relationship between *Pitx3* and *En1* during the development of mdDA neurons, we inter-crossed *En1*^*tm1Alj/+*^ animals with *Pitx3*^*gfp/gfp*^ animals to create double *En1*^*tm1Alj/tm1Alj*^*;Pitx3*^*gfp/gfp*^ (En1KO;Pitx3GFP/GFP) animals. First of all, in order to create the desired En1KO;Pitx3GFP/GFP genotype, we initially bred an 'intermediate genotype' *En1*^*tm1Alj/+*^*;Pitx3*^*gfp/gfp*^ (Fig 1A). These animals were bred to generate litters that included *En1*^*+/+*^*;Pitx3*^*gfp/gfp*^ (En1WT;Pitx3GFP/GFP), En1^*tm1Alj/+*^;Pitx3^*gfp/gfp*^ (En1Het;Pitx3GFP/GFP) and *En1*^*tm1Alj/tm1Alj*^*;Pitx3*^*gfp/gfp*^ (En1KO;Pitx3GFP/GFP). En1Het;Pitx3GFP/GFP animals were blind and smaller, due to the absence of *Pitx3* as described previously [29,31]. As a consequence the minimum weight to conceive was reached later, i.e. after four months compared to 6–8 weeks in control C57BL/6J animals housed and bred in our animal facility (Table 1). Moreover, upon birth En1KO;Pitx3GFP/GFP animals were smaller and skinny, and displayed perinatal lethality and were therefore isolated at P0 at the latest. Furthermore, litter size was relatively small and the En1KO;Pitx3GFP/GFP genotype presented itself significantly less in litters than expected based on Mendelian distribution (Fig 1B). Due to these breeding constrains we were only able to obtain and examine one litter at P0, and four litters at E14.5 (n = 3 for FACS analysis, n = 1 for anatomical descriptions).

**Table 1:** Breeding constrains in creating double En1KO;Pitx3GFP/GFP animals.

	En1Het;Pitx3GFP/GFP X En1Het;Pitx3GFP/GFP	C57BL/6J (wild-type)^§^
Average age of pregnancy	4.4 months (± 0.5)	6–8 weeks
Average weight at conception	22.3 grams (± 1.0)	20 grams
Average litter size	5.8 (± 0.7) pups	8 pups

^§^ Based on observations from our animal facility.

The earliest studies on *En1*-ablated mice reported perinatal lethality, due to cerebellar ablation [32], however our group and others reported that this phenomenon may be circumvented by back-crossing the original 129/Sv line to a C57BL/6J background [13,33]. To determine the influence of the mouse-model background in these double En1KO;Pitx3GFP/GFP animals, we first examined the cytoarchitecture of the mid- and hindbrain area (using a DAPI staining). At E14.5 the *anlage* of the cerebellum is clearly present in En1WT;Pitx3GFP/GFP and En1Het;Pitx3GFP/GFP animals (Cb, Fig 1C), whereas the *anlage* was absent in En1KO;Pitx3GFP/GFP animals (asterisk, Fig 1C). These morphological changes are highly similar to the original En1KO. Even though the *En1*^*tm1Alj/+;*^*Pitx3*^*gfp/gfp*^ animals were back-crossed unto C57BL/6J animals this cerebellar ablation suggests that the current analysis of the double loss of *En1* and *Pitx3* is influenced by the genetic background effects as described for the original *En1*-mutation [32].

### The cytoarchitecture of the developing mdDA area is affected in *En1*/*Pitx3* double mutants

**Fig 2:**
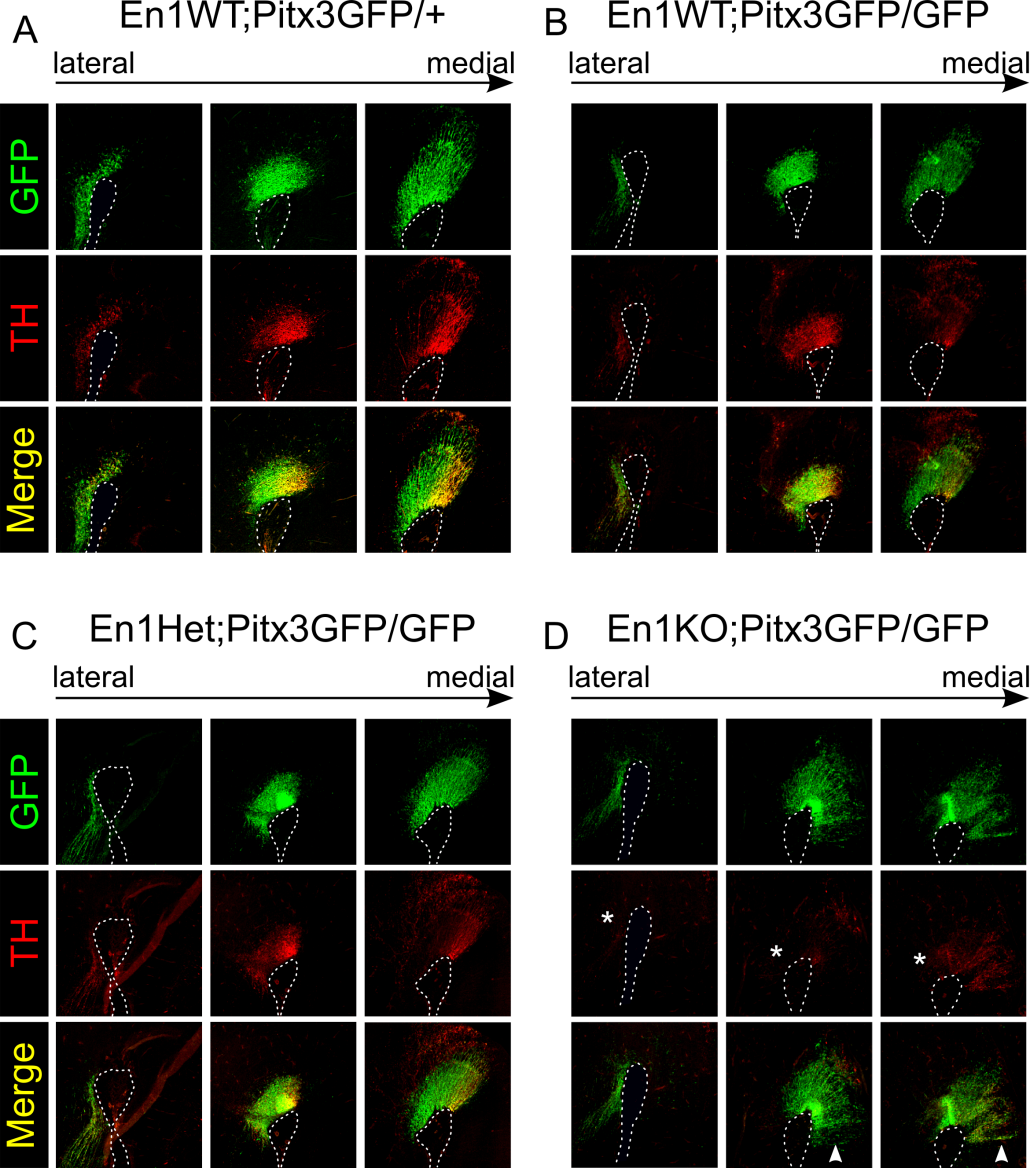
Analysis of TH and GFP expression in multiple En1-/Pitx3-mutants at E14.5. (A) Immunohistochemistry of GFP and TH in different sections from medial to lateral encompassing the mdDA neuronal pool in Pitx3GPF/+ animals. (B-D) Same setup as described for A for (B) En1WT;*Pitx3*GFP/GFP, (C) En1Het;*Pitx3*GFP/GFP, and (D) En1KO;*Pitx3*GFP/GFP animals (matching sections with A). (D) Asterisk indicate the loss of TH immunoreactivity in more medial sections. Arrowheads indicate the presence of ectopic mdDA neurons.

To examine the combined role of *Pitx3* and *En1* in the cytoarchitecture of the mdDA system, we analysed TH protein and GFP expression at E14.5 in En1WT;Pitx3GFP/GFP, En1Het;Pitx3GFP/GFP, En1KO;Pitx3GFP/GFP litter mates and in an En1WT;Pitx3GFP/+ controls from a different litter. The phenotypical changes that have been previously ascribed to the single En1KO or the single Pitx3KO mutants are visible in the double En1KO;Pitx3GFP/GFP animal at E14.5 (Fig 2). First of all, similar to both single knock-outs TH protein was strongly diminished in lateral mdDA regions (asterisk, Fig 2D). Noteworthy, in the En1KO;Pitx3GFP/GFP animals the loss of TH immunoreactivity is more severe, as TH is also lost in more medial sections (compared to all other genotypes, Fig 2). Second, in (para)medial sections the presence of ectopic mdDA neurons (eDA neurons) was evident, which was recently attributed to changes in the maintenance of the Isthmic Organizer, in absence of *En1* [30]. Note that these eDA neurons are marked by both by TH and GFP expression (arrow head, Fig 2D). Finally, using GFP as a marker for mdDA neurons in these models, it appears that in the medial sections fewer mdDA neurons are present in absence of both *Pitx3* and *En1* (Fig 2D), compared to all other genotypes (Fig 2A–2C). Together, these data suggest that in absence of both *Pitx3* and *En1* the survival and/or generation of mdDA neurons is more severely affected, as are the programming defects in terms of number of mdDA neurons that contain TH protein.

**Fig 3:**
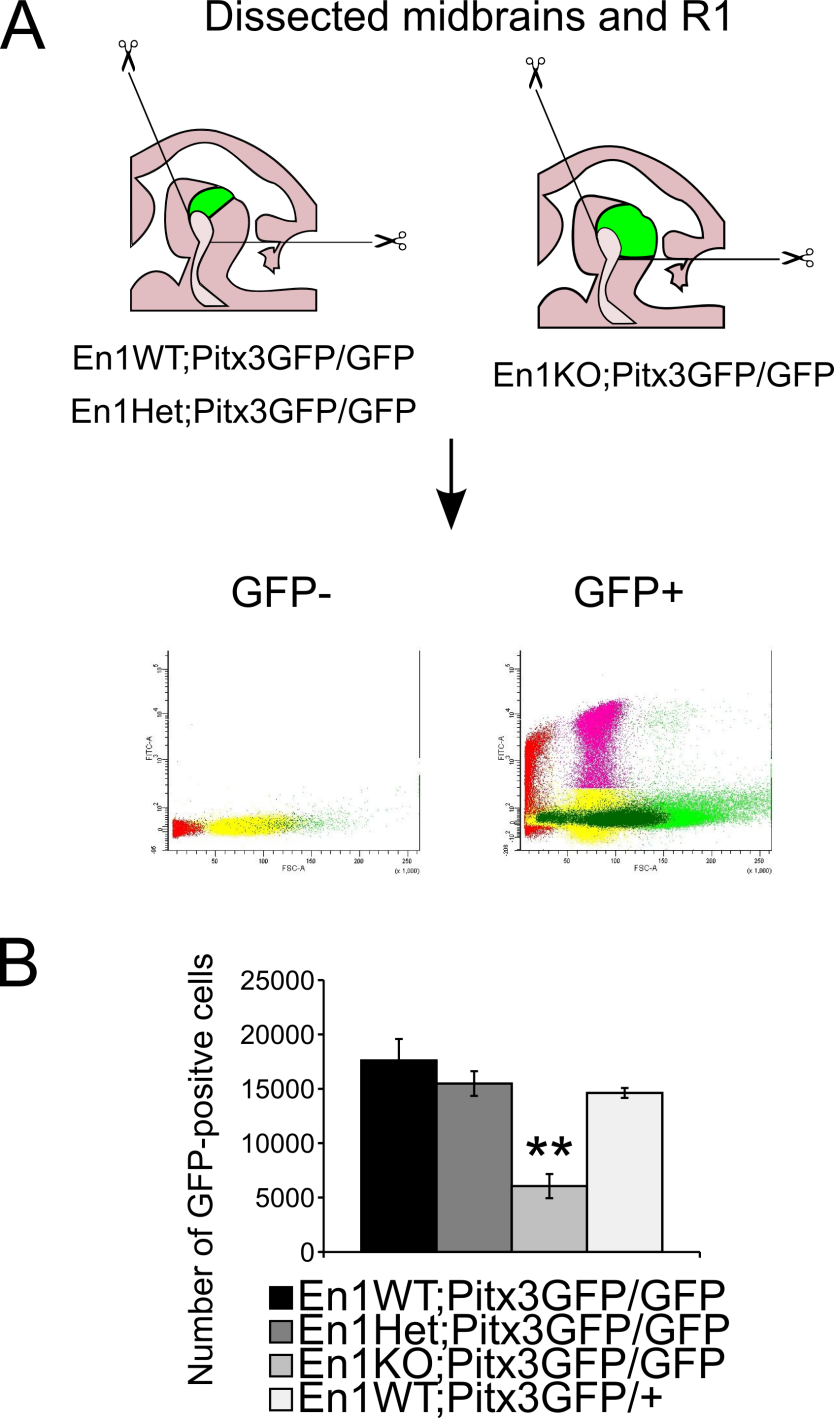
Quantification of the number of GFP-positive neurons present in the midbrains of multiple *En1*-/*Pitx3*-mutants at E14.5. (A) Schematic representation of the isolation of midbrain and R1, and subsequent FAC-sorting setup at E14.5 to be used for quantification of number of GFP-positive neurons. (B) At E14.5, ~15000 GFP-positive mdDA neurons were sorted from control, En1WT;Pitx3GFP/GFP and En1Het;Pitx3GFP/GFP animals. In contrast, only ~5000 GFP-positive mdDA neurons were present in the En1KO;Pitx3GFP/GFP midbrain/R1 (** = P<0.01, n = 3/4).

To get an objective measurement of the number of GFP-positive mdDA neurons that are still present in the midbrain of double En1KO;Pitx3GFP/GFP animals, we used the presence of GFP under the promoter of *Pitx3* to selectively sort GFP-positive mdDA neurons [22,29]. Previous work from our group revealed the eDA neurons that are present in the rostral hindbrain of *En1*-ablated animals are indistinguishable from mdDA neurons [30], thus we dissected midbrains and rostral hindbrains of litter mates En1WT;Pitx3GFP/GFP, En1Het;Pitx3GFP/GFP, En1KO;Pitx3GFP/GFP and En1WT;Pitx3GFP/+ control animals from a different litter, at E14.5 (Fig 3A). This approach enabled us to quantify the number of GFP-positive mdDA neurons that are present in the four different genotypes. At E14.5, ~15000 GFP-positive mdDA neurons were sorted from control animals, and this number did not change significantly in En1WT;Pitx3GFP/GFP and En1Het;Pitx3GFP/GFP animals. However, in the absence of both *En1* and *Pitx3* this number decreased 3-fold to ~5000 GFP-positive mdDA neurons (P<0.01, n = 3/4, Fig 3B). These data substantiated our observations that in the double En1KO;Pitx3GFP/GFP animal fewer mdDA neurons are present at E14.5, compared to the other analysed genotypes.

### *En1* and *Pitx3* activate the dopaminergic program synchronously

**Fig 4:**
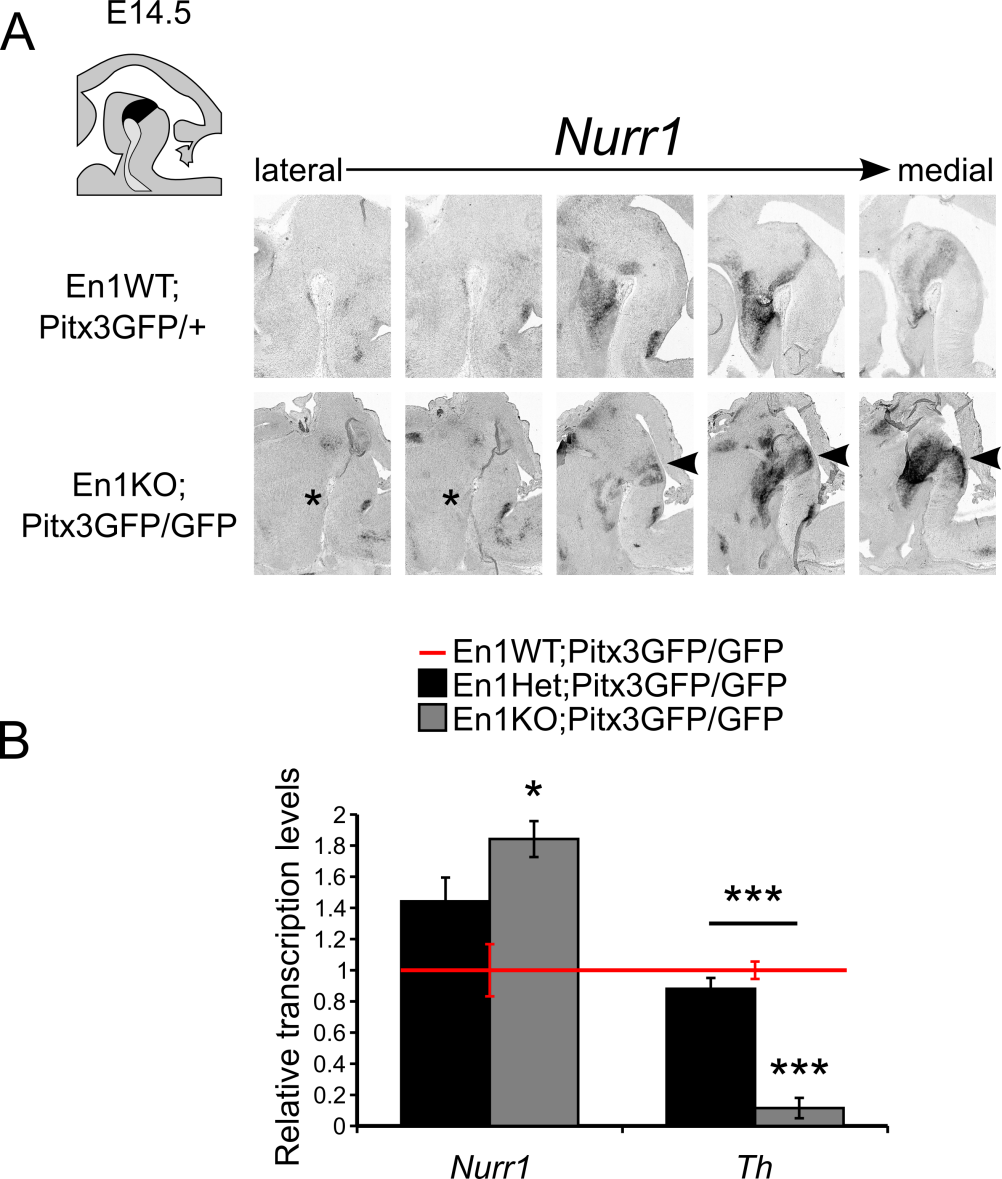
*Nurr1*-positive mdDA neurons are present in double En1KO;Pitx3GFP/GFP animals at E14.5. (A) Schematic, sagittal section of the embryonic mouse brain, mdDA area is indicated in black. Lateral to medial sections of *in situ* hybridization experiments for *Nurr1* at E14.5 in En1WT;Pitx3GFP/+ and En1KO;Pitx3GFP/GFP midbrains. Asterisk indicates diminished expression of *Nurr1* in lateral sections, whilst in (para)medial sections *Nurr1* is still present in the midbrain and is ectopically extended caudally (arrowheads). (B) Quantitative PCR on FAC-sorted mdDA neurons demonstrates elevated level of *Nurr1* in the En1KO;Pitx3GFP/GFP midbrain, compared to En1WT;Pitx3GFP/GFP (* = P<0.05, n = 3/4). The expression of *Th* expression is significantly down-regulated in the En1KO;Pitx3GFP/GFP midbrain, compared to En1WT;Pitx3GFP/GFP (*** = P<0.01, n = 3/4).

Both *En1* and *Pitx3* are pivotal players for the proper induction of the molecular dopaminergic profile, as illustrated by the loss of expression of *Nurr1*-targets in the single *En1KO* and the single *Pitx3KO* animal [13,31]. Thus, we aimed to determine which parts of the molecular dopaminergic machinery were still intact in the absence of both *En1* and *Pitx3*. We used *in situ* hybridization to investigate the expression pattern of *Nurr1* in En1WT;Pitx3GFP/+, and En1KO;Pitx3GFP/GFP animals (Fig 4). This analysis revealed regional alterations: in lateral sections of the double En1KO;Pitx3GFP/GFP a small area in the ventral diencephalon positive for *Nurr1* transcript cannot be detected (Fig 4A, asterisk), whilst in (para)medial sections *Nurr1* was present in a changed level and architecture in midbrain and ectopically extend caudally (arrowhead, Fig 4A). In order to further investigate the programming consequences of *En1*- and *Pitx3-*ablation we sorted GFP-positive neurons and subjected the RNA in those cells to qPCR analyses. Since the chosen breeding scheme did not allow for the presence of a control Pitx3GFP/+ litter mate (Fig 1), we elected to use the En1WT;Pitx3GFP/GFP genotype as a point of reference to analyze relative transcript levels, as genome wide expression analysis of En1WT;Pitx3GFP/GFP animals compared to control have already been published [27]. These analyses revealed that *Nurr1* transcript levels were significantly increased in En1KO;Pitx3GFP/GFP mdDA neurons compared to En1WT;Pitx3GFP/GFP animals (Fig 4B, P<0.05, n = 3–4).

Furthermore, we investigated the transcript level of *Th*, as we previously established that in both the single Pitx3KO and the single En1KO, *Th* transcript is significantly diminished [13,27,31]. We observed an even further drop in *Th* transcript expression in En1KO;Pitx3GFP/GFP mdDA neurons compared to both En1WT;Pitx3GFP/GFP and En1Het;Pitx3GFP/GFP mdDA neurons (Fig 4B, P<0.001, n = 3–4). This observation is in line with the loss of TH protein, identified in immunohistochemistry experiments at E14.5 (Fig 3). Together these data confirm that *Pitx3* and *En1* act in synchrony to induce the *Th* gene in mdDA neurons, independent of *Nurr1*.

### *En1* and *Pitx3* program mdDA neurons to a specific rostral/caudal subtype

**Fig 5:**
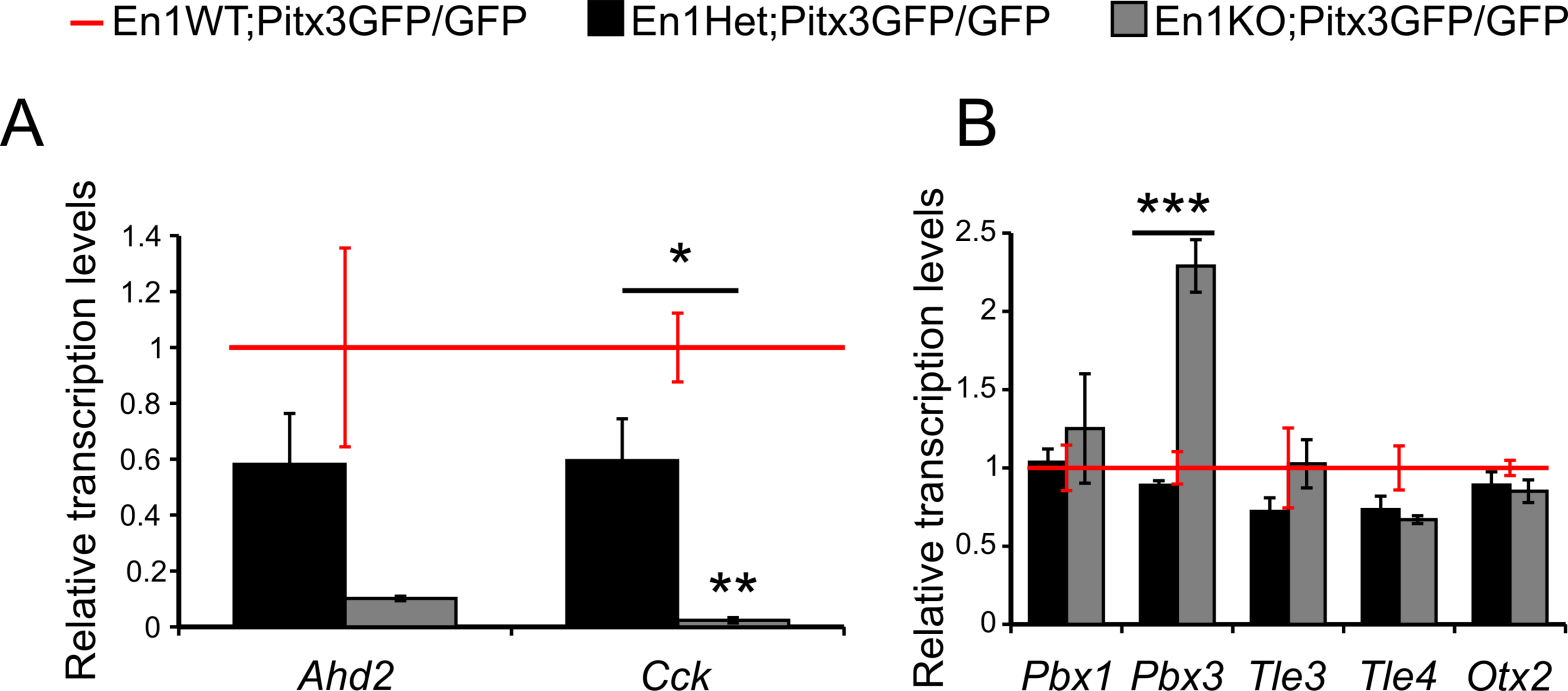
Quantitative PCR analysis of mdDA neurons in double En1KO;Pitx3GFP/GFP animals at E14.5. (A) Quantitative PCR demonstrates no further loss of *Ahd2* expression in En1KO;Pitx3GFP/GFP animals compared to En1WT;Pitx3GFP/GFP (P>0.05, n = 3/4). Significant loss of *Cck* expression in both the En1Het;Pitx3GFP/GFP (P<0.05, n = 4) and En1KO;Pitx3GFP/GFP animals (P<0.01, n = 3/4), compared to En1WT;Pitx3GFP/GFP. (B) Quantitative PCR demonstrates no changes in *Pbx1*, *Tle3*, *Tle4* and *Otx2* expression in En1KO;Pitx3GFP/GFP animals compared to En1WT;Pitx3GFP/GFP (P>0.05, n = 3/4). Significantly increased expression of *Pbx3* in En1KO;Pitx3GFP/GFP animals, compared to both the En1Het;Pitx3GFP/GFP and En1WT;Pitx3GFP/GFP animals (*** = P<0.01, n = 3/4).

The mdDA neurons that make up the SNc are located in the rostrolateral midbrain during development (E14.5) and express *Ahd2*, while the mdDA neurons that will develop into the VTA express *Cck* and are located caudomedially [13,26]. Previous research revealed that the absence of either *Pitx3* or *En1* affects each subset differently; *En1* initially promotes *Pitx3* in the rostral subset, but is subsequently repressed by *Pitx3*. This was illustrated by an increase of *En1* in the Pitx3KO, and the loss of *Pitx3* in the rostral subset in the En1KO. It has been proposed that *Pitx3* and *En1* play an interrelated role in the programming of the rostral versus caudal subset. To refine our understanding of the regional-specific actions of *En1* and *Pitx3*, we examined the expression levels of subset-specific markers *Ahd2* and *Cck* in double En1KO;Pitx3GFP/GFP animals at stage E14.5 of development (Fig 5). In absence of both *En1* and *Pitx3* the expression of *Ahd2* is not significantly decreased further relative to En1WT;Pitx3GFP/GFP mdDA neurons (Fig 5A, P>0.05). It is worth pointing out that in absence of solely *Pitx3*, the expression of *Ahd2* is already severely affected [26], which is equally true for *En1*-null mice [13]. These data support the notion that *En1* and *Pitx3* both promote *Ahd2* expression in unison: *Pitx3* most likely in a direct manner (binding to the *Ahd2*-promotor [26]), while *En1* could promote *Ahd2* expression either directly or via its control on *Pitx3* [13] or both.

In contrast, *En1* and *Pitx3* exert opposing effects on the caudal-subset programming, i.e. *Cck* expression. In absence of *Pitx3*, *Cck* is up-regulated [27] whereas *Cck* expression is lost in absence of En1 [13]. Thus, we queried whether the combined absence of *En1* and *Pitx3* would either restore *Cck* expression (through a loss of *Pitx3*-mediated repression) or inhibit its expression (due to the loss of *En1*-mediated activation). Indeed, double En1KO;Pitx3GFP/GFP animals display a significant loss of *Cck* compared to the single Pitx3KO (P<0.01, Fig 5A). Furthermore, the graded decline of *Cck* transcript expression with the loss of each *En1* allele reveals its dose-dependent sensitivity to the (in)activation of *En1*, independent of *Pitx3* (P<0.05, Fig 5A). This suggest that *Cck* is only dependent on its activation by *En1*.

In addition to *Pitx3*, *En1* and *Nurr1*, other transcription factors have been shown to contribute to the development of (a subset of) mdDA neurons [34]. In order to better characterize the subset of mdDA neurons that is still present in the midbrain in absence of *Pitx3* and *En1*, we elected to investigate the expression levels of certain relevant transcription factors. To start, we previously established clear expression of several members of the Tle/Groucho family, known interactors of *En1* and *Pitx3*, within mdDA neurons. In addition, we revealed that the expression of *Pbx1* and *Tle3* is enriched in caudal mdDA neurons, whereas *Pbx3* and *Tle4* are rostrally enriched. Finally, we reported that the expression levels of *Pbx1*, *Pbx3* and *Tle3* are dependent on *Pitx3* activity, [13]. Thus, we examined their relative expression in the absence of both Pitx3 and *En1*. Here, we report no changes in the expression levels of *Pbx1*, *Tle3* and *Tle4* between En1WT;Pitx3GFP/GFP and En1KO;Pitx3GFP/GFP (Fig 5B, P>0.05). Interestingly, the relative expression of *Pbx3* is significantly increased in mdDA neurons of En1KO;Pitx3GFP/GFP, compared to both En1WT;Pitx3GFP/GFP and En1Het;Pitx3GFP/GFP (Fig 5B, P<0.001, n = 3–4). Since the expression of *Pbx3* is diminished in the single Pitx3KO, its remarkable increase in the double En1KO;Pitx3GFP/GFP suggests that the absence of *En1* has lifted an inhibition of *En1* on *Pbx3* expression.

Finally, we investigated the expression of *Otx2*, as previous research has determined that it plays an important role in the survival and programming of (a subset) of mdDA neurons [35,36]. We report no changes in the expression levels of *Otx2* between En1WT;Pitx3GFP/GFP and En1KO;Pitx3GFP/GFP (Fig 5B, P>0.05). Thus, the remaining population of mdDA neurons in the En1KO;Pitx3GFP/GFP is positive for *Otx2*, a transcription factor that is known to contribute to the survival of mdDA neurons during adverse conditions [37] and mainly present in a caudal subset of mdDA neurons.

### Loss of both *En1* and *Pitx3* affects survival of mdDA neurons upon P0

**Fig 6:**
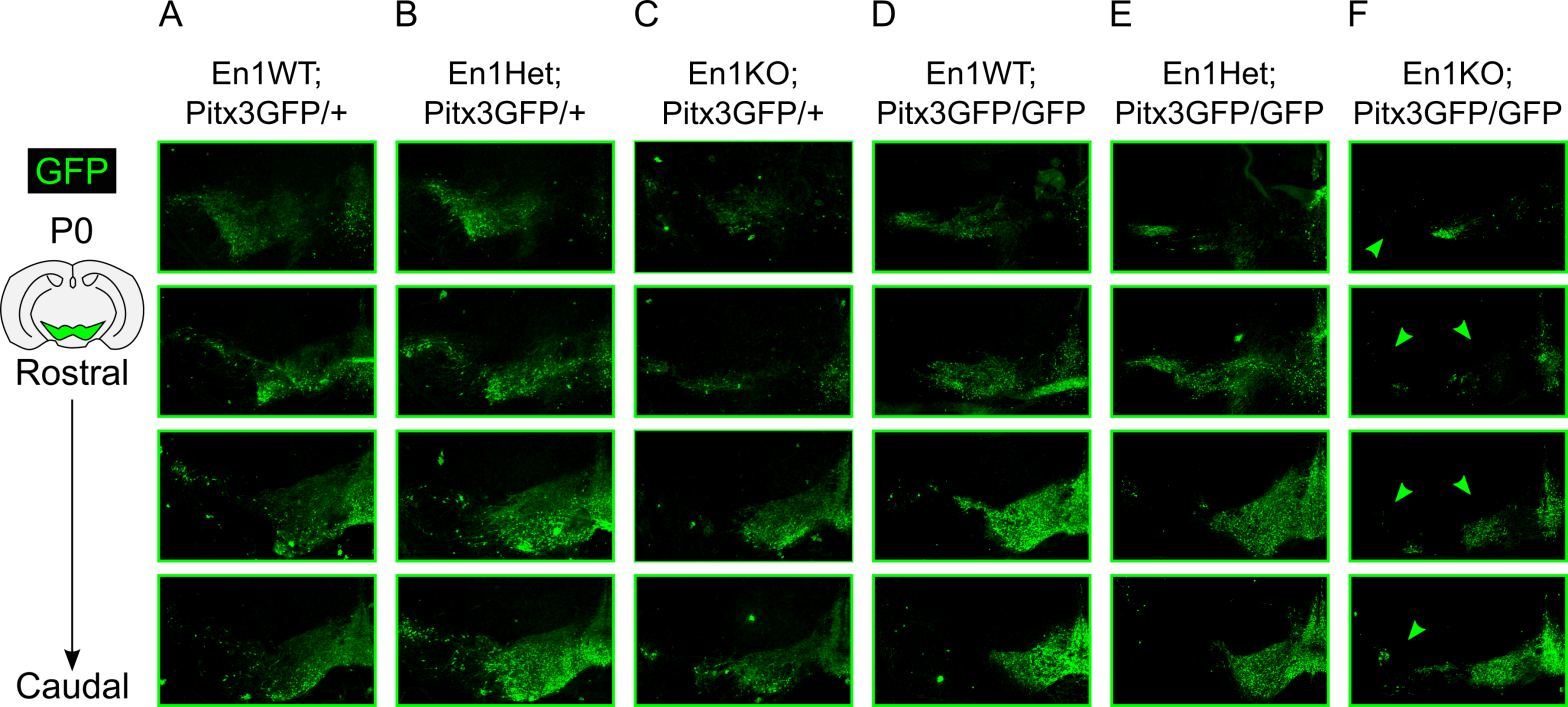
Qualitative analysis of GFP expression in multiple *En1*-/*Pitx3*-mutants at P0. Schematic, coronal section of the adult mouse brain, mdDA area is indicated in green. (A) Immunohistochemistry of GFP in different sections from rostral to caudal encompassing the mdDA neuronal pool in Pitx3GPF/+ animals. (B-F) Same setup as described for A for (B) En1Het;Pitx3GFP/+, (C) En1KO;Pitx3GFP/+, (D) En1WT;Pitx3GFP/GFP, (E) En1Het;Pitx3GFP/GFP, and (F) En1KO;Pitx3GFP/GFP animals (matching sections with A). Arrowheads indicate the loss of GFP-positive mdDA neurons in the SNc and VTA region (n = 1).

Single Pitx3KO animals are characterized by an embryonic loss of *Ahd2* and the post-natal loss of the SNc, while in the En1KO both *Ahd2* and *Cck* are lost embryonically, which is accompanied by a post-natal defect in both the SNc and VTA. We just established that the midbrain of double En1KO;Pitx3GFP/GFP animals contains ~60% less GFP-positive mdDA neurons (E14.5), and that these mdDA neurons do not express their characteristic rostral and caudal marks. In order to obtain a better understanding of the (combined) role of *En1* and *Pitx3* in survival of mdDA neurons, we examined the cytoarchitecture through GFP immunohistochemistry at the latest possible time-point (Fig 6).

Due to the described breeding difficulties we were only able to obtain one double *Pitx3*/*En1*-ablated mouse at P0, thus the immunohistochemistry of the GFP is a qualitative description of the cytoarchitecture, in which we included six different genotypes: En1WT;Pitx3GFP/+; En1Het;Pitx3GFP/+; En1KO;Pitx3GFP/+; En1WT;Pitx3GFP/GFP; En1Het;Pitx3GFP/GFP; En1KO;Pitx3GFP/GFP. First, the largest differences in cytoarchitecture of the mdDA area are observed when at least two alleles of one gene are ablated i.e. the En1KO;Pitx3GFP/+, the single Pitx3KO, the En1Het;Pitx3GFP/GFP, and finally the En1KO;Pitx3GFP/GFP (arrowheads, Fig 6C–6F). Second, the absence of *Pitx3* mainly affects the SNc, as described in great detail before (Fig 6D; [29,31,38–40]. Third, in contrast, the absence of two alleles of *En1* seemed to target the GFP-positive mdDA neurons of both SNc and VTA (Fig 6C, 6D and 6F), which has also been previously described (albeit at a later age)[13]. Finally, double En1KO;Pitx3GFP/GFP animals display severe defects in the entire mdDA area: The SNc is mostly lost, except for a small spot of GFP-positive cells, and the VTA is much smaller compared to any other genotype (arrowheads, Fig 6F). Although the data on the Pitx3/En1 double ablated animals is only present at an N = 1 we conclude that the data substantiate previous data, in the sense that *En1* and *Pitx3* act together in generating the mdDA neuronal pool and that a small portion might not not depend on both transcription factors for their generation and survival (until P0).

## Discussion

### Genetic background influences the phenotype of En1KO;Pitx3GFP/GFP animals

In the current manuscript we aimed to refine our understanding of the effects of *Pitx3* and *En1* activity on each other, and the development of mdDA neurons. Our group and others have previously investigated the single Pitx3KO [29,31,38–40] and the single En1KO [13,30] in great detail, and we elected to include that data in our study to allow us to draw comparisons between the single Pitx3KO, the single En1KO, and the double En1KO;Pitx3GFP/GFP. To start, upon birth En1KO;Pitx3GFP/GFP animals were smaller, and skinny, and displayed perinatal lethality. Furthermore, we observed that the cerebellar *anlage* was lost in the double En1KO;Pitx3GFP/GFP animal (Fig 1). This phenotype is identical to the En1-mutation in the 129/Sv mouse background [32]. Both *Pitx3*^*gfp/gfp*^ animals and *En1*^*tm1Alj/+*^ animals were back-crossed onto a C57BL6/J background, however it takes several generations of back-crossing to completely neutralize the 129/Sv mouse background, and generate viable En1KO animals [13,30,33]. We thus hypothesize that the perinatal lethality is most likely the consequence of the presence of the 129/Sv mouse background. Consequently, we excluded viability as a criterion in our analyses in double En1KO;Pitx3GFP/GFP animals.

### Absence of both *En1* and *Pitx3* severely affects mdDA neurons

We examined the cytoarchitecture of several *Pitx3*-/*En1*-mutants at E14.5 and P0, and quantified the number of GFP-positive mdDA neurons in the midbrain at E14.5. Both the single Pitx3KO and the single En1KO are characterized by diminished expression of *Nurr1*/*Pitx3*/*En1*-targets during embryonic development, but mdDA neuronal cell loss is not yet present at E14.5 [31,39]. Both the single En1KO and the Pitx3KO demonstrate normal expression of *Nurr1* at E12.5 suggesting that the neurogenesis of early post-mitotic mdDA neurons occurs correctly [13]. When examining double En1KO;Pitx3GFP/GFP animals it is important to take into account that until E11.5 the development of mdDA neurons in the single En1KO and the double En1KO;Pitx3GFP/GFP animal are identical. Still, we demonstrate that the double loss of *En1* and *Pitx3* does not merely mimic the En1KO phenotype, but represents a more severe phenotype. This suggest that the combined loss of *Pitx3* and *En1* (and their subsequent targets) after E11.5 is causative to the significant cell loss observed at E14.5 in double En1KO;Pitx3GFP/GFP animals compared to controls and Pitx3KOs. Previous data from single En1KO- and Pitx3KO animals revealed that both transcription factors control the expression of a broad myriad of genes and other transcription factors [13,22,31,41]. After E11.5, the combined loss of any of such targets and their subsequent molecular pathways contribute to the phenotype of double En1KO;Pitx3GFP/GFP animals. Taken together, the diminished number of mdDA neurons in double En1KO;Pitx3GFP/GFP animals may find its origin in an absence of survival factors, or an absence of proper dopaminergic differentiation. In sum, both *En1* and *Pitx3* are important for the survival of developing mdDA neurons, and thus the size of the mdDA neuronal pool.

### Does a compensatory mechanism of transcription factors contribute to the survival of (a subset of) mdDA neurons?

The importance of *Nurr1* in the embryonic development of mdDA neurons has been clearly established for years [21,22,24]. The expression of *Nurr1* is thought to be independent of both *Pitx3* and *En1*, as expression levels and patterns of *Nurr1* are unchanged in single Pitx3KO and single En1KO animals [13,22]. Still, we report here a significant increase in *Nurr1* transcript levels in mdDA neurons of double En1KO;Pitx3GFP/GFP animals. This could suggest that through the double absence of *Pitx3* and *En1* a regulatory inhibition is lifted. Alternatively, this might suggest that via a still unknown mechanism, the expression of *Nurr1* is boosted as a possible compensatory mechanism, to promote the survival and functioning of the remaining mdDA neurons.

Moreover, we previously demonstrated that the expression of *Pbx3* is enriched in rostral embryonic mdDA neurons, adult neurons of the SNc and is dependent on activation by *Pitx3* [13,24]. Here we report a significant increase in *Pbx3* expression in mdDA neurons in the absence of both *Pitx3* and *En1* (Fig 5). Even though *Pbx3* did not surface as a target of *En1* activity in genome wide expression analysis on *En1*-ablated embryonic midbrains [13], these data could suggest that *En1* represses the expression of *Pbx3* during wild type embryonic development. This aligns with the hypothesis that *En1* favours the programming of caudal mdDA neurons. Furthermore, other authors have speculated on a possible redundancy between *Pbx1* and *Pbx3* [42–44]. This notion was recently strengthened by an elaborate study which investigated several *Pbx1*/*Pbx3*-mutants, and revealed that a conditional *Pbx1*;*Pbx3* knock-out resulted in more severe loss of TH-positive neurons than either the single *Pbx1* or *Pbx3* knock out mouse. In fact, *Pbx3* levels were increased in the *Pbx1* null-mouse [45]. In similar line of thought, the elevated levels of *Pbx3* in double *Pitx3*/*En1*-ablated mdDA neurons that we report in the current study (Fig 5B) could also act as a compensatory mechanism, and contribute to the survival of the small group of remaining mdDA neurons.

Finally, we confirmed that the presence of *Otx2* in the remaining mdDA neurons of the double En1KO;Pitx3GFP/GFP animal, is not different from the *Otx2* expression in single Pitx3KO animals (Fig 5B). Furthermore, *Otx2* is known marker of a subset of VTA neurons, and has been shown to promote survival of mdDA neurons in multiple adverse conditions (e.g. MPTP toxicity and *En1*-haplo-insufficiency) [35,37]. Thus, it might be possible that the combined presence of *Nurr1*, *Pbx3* and *Otx2* contribute to the (temporary) survival of a small subset of mdDA neurons in double En1KO;Pitx3GFP/GFP animals.

### Relative levels of *Pitx3* and *En1* determine rostral-caudal identity and the size of the embryonic mdDA neuronal pool

**Fig 7:**
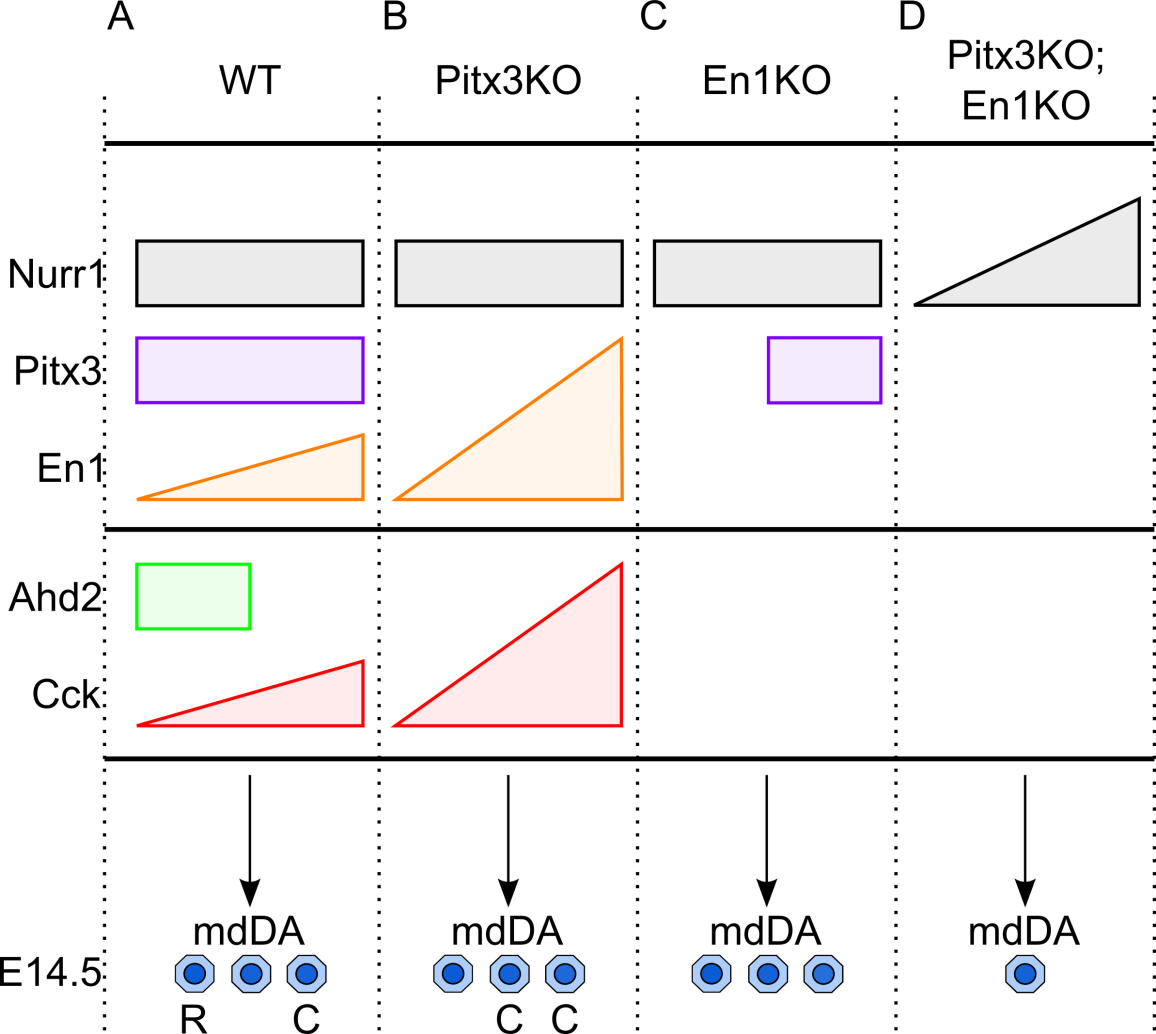
Schematic representation of roles of *En1* and *Pitx3* in the programming of the rostral-caudal identity of mdDA neurons. (A) In wild-type midbrain *Nurr1* initiates the development of mdDA differentiation, *Pitx3* promotes *Ahd2* expression and represses *En1* in rostral midbrain, whilst *En1* promotes *Pitx3* and *Cck* expression. The mdDA neuronal pool includes rostral-coded and caudal-coded neurons. (B) In *Pitx3*-ablated animals *Nurr1* initiates the development of mdDA differentiation, though *Ahd2* expression is lost, and the inhibition of *Pitx3* on *En1* is lifted, thus *En1* and subsequently *Cck* are up-regulated. The mdDA neuronal pool includes only caudal-coded neurons. (C) In *En1*-ablated animals *Nurr1* initiates the differentiation of mdDA progenitors, though *Cck* expression is lost, and *Pitx3* expression in the rostral midbrain is not initiated, thus *Ahd2* expression is lost as well. The mdDA neuronal pool includes only non-coded neurons. (D) In double En1KO;Pitx3GFP/GFP animals elevated levels *Nurr1* promotes the differentiation of mdDA progenitors, though *Cck* and *Ahd2* are lost.

During wild-type development the transcriptional regulation of *Nurr1*, *Pitx3* and *En1* induce mdDA neurons. Two important subsets that can be identified at E14.5 within the total and divers pool of mdDA neurons are rostral (R) mdDA neurons that express *Ahd2* and become SNc neurons, and caudal (C) mdDA neurons (*Cck*+) that develop into the VTA (Fig 7A). In *Pitx3*-deficient mice unaltered *Nurr1* expression represents the normal initiation of mdDA progenitors, however *Ahd2* expression is lost. Moreover, the inhibitory regulation of *Pitx3* on *En1* is released, and therefore *En1* expression is significantly increased, resulting in the concomitant up-regulation of *Cck* expression [13,26]. These changes in the programming of mdDA neurons result in the absence of rostral-coded mdDA neurons, and an over-representation of caudally programmed mdDA neurons (Fig 7B). In *En1*-null mice, unchanged *Nurr1* expression reveals the initiation of mdDA progenitors, though the expression of *Pitx3* and *Ahd2* is affected in the rostral midbrain, and the expression of *Cck* is lost as well [13]. Together these deficiencies result in the presence of mdDA neurons that are devoid of the rostral-caudal markers which might be a representation of the loss of the correct programming of the rostral and caudal subset (Fig 7C). Finally, in absence of *Pitx3* and *En1* mdDA progenitors are still initiated, revealed by the (elevated) presence of *Nurr1*, but significantly fewer GFP-positive mdDA neurons are present at E14.5. Furthermore, the expression of *Ahd2* and *Cck* is completely lost (Fig 7D). In sum, the current study supports the hypothesis that *Pitx3* primarily promotes the specification of mdDA neurons into a SNc neuron, whereas *En1* drives mdDA neurons towards a VTA fate and may be higher-up in the hierarchy of the transcriptional programming [34]].
